# Recent Advances in Discotic Liquid Crystal-Assisted Nanoparticles

**DOI:** 10.3390/ma11030382

**Published:** 2018-03-05

**Authors:** Ashwathanarayana Gowda, Sandeep Kumar

**Affiliations:** Soft Condensed Matter Group, Raman Research Institute, C.V. Raman Avenue, Sadashivnagar, Bangalore 560 080, India; ashwathgowda@rri.res.in

**Keywords:** discotic liquid crystal, columnar phase, nanoparticles, quantum dots, graphene, nanocomposites

## Abstract

This article primarily summarizes recent advancement in the field of discotic liquid crystal (DLC) nanocomposites. Discotic liquid crystals are nanostructured materials, usually 2 to 6 nm size and have been recognized as organic semiconducting materials. Recently, it has been observed that the dispersion of small concentration of various functionalized zero-, one- and two-dimensional nanomaterials in the supramolecular order of mesophases of DLCs imparts negligible impact on liquid crystalline properties but enhances their thermal, supramolecular and electronic properties. Synthesis, characterization and dispersion of various nanoparticles in different discotics are presented.

## 1. Introduction

### 1.1. Discotic Liquid Crystals

The self-assembly of appropriately functionalized disc-like molecules prompts to the formation of discotic liquid crystals (DLCs) [1,2]. DLCs are unique nanostructures with exceptional optoelectronic properties. The concept of mesomorphism in organic materials was discovered more than a century ago when Austrian botanist, Friedrich Reinitzer, in 1888 observed the dual melting point of cholesterol benzoate [3]. From then until 1977 it was observed that only rod-shape or calamitic molecules form liquid crystals. In 1977 Chandrasekhar et al. reported “that not only compound with rod-like molecular shape but also disc-like anisotropic molecular structures can exhibit mesomorphic behavior” [4]. This family of liquid crystalline materials is presently known as DLCs. Generally, these materials consist of flat or nearly flat central aromatic disc-shape core, substituted by more than three flexible aliphatic carbon chains. Such functionalized disc-shape molecules spontaneously self-assemble into nematic, columnar or lamellar mesophases [2]. A large number of DLCs are derived from polycyclic aromatic hydrocarbons possessing strong π–π interactions among the aromatic cores and, therefore, spontaneously self-organized one on the top of another to form columnar stacks. In general, DLCs with large π-conjugated aromatic rings enhance the columnar stability, supramolecular order and high charge carrier mobility due to extended π-orbital overlap. About 95% of DLCs exhibit a columnar mesophase and only about 5% of DLCs exhibit a discotic nematic (N_D_) phase. In the discotic nematic phase, discotic molecules have only directional order but not long range positional order while in columnar phases, molecules possess both orientational and positional orders. Both the mesophases formed by discotic molecules are extremely important from device application perspectives. Discotic nematic LCs have been used as optical compensation films to enhance the viewing angle in liquid crystal display (LCDs) [5] and their use as active component in wide-viewing LCDs applications has also been sought [6,7,8,9]. 

In the columnar mesophase, discotic mesogens tend to pack firmly, like the molecular arrangement in a solid substance, and the intracolumnar (core-core) distance, depending on the molecular structure of central core, is typically in the order of 0.35 nm while intercolumnar (neighboring columns) distance is usually 2–4 nm, contingent upon the length of flexible alkyl chains. DLCs are organic materials and insulators in their authentic state; however, they can be made to charge transport materials by producing charges chemically or photochemically. Various dopants, e.g., I_2_, Br_2_, trinitrofluorenone, aluminum chloride, ferrocinium ions etc., have been used to investigate the electrical conducting properties of these systems [10,11,12,13,14]. For example, electrical conductivity of triphenylene based DLC, hexahexylthiotriphenylene (HHTT) increased by a factor of 10^6^ or more when mixed with a very low concentration of trinitrofluorenone (TNF) (0.62 mol%) [14]. The electrical conductivity (direct current DC) of DLCs nanocomposites shows appreciable anisotropy, σ_‖_/σ_⊥_ ≥ 10^10^. Moreover, increasing π-conjugation in many DLCs derived from cyclic aromatic hydrocarbons enhances the charge carrier mobility. This research topic has been described in many recent review articles e.g., [15,16,17]. The close stacking of molecules in columnar phase can enhance hasty roadway for the transfer of produced charges via hopping from one discotic molecule to another. The charge transport mobility in these discotic materials is expected to be quasi-1-dimensional due to conducting part in the disc like molecules surrounding by the insulating aliphatic chains. Thus, the columns can be depicted as molecular wires. Inherent charge transport mobilities up to 0.71 cm^2^·V^‒1^·s^‒1^ along the columns have recently been observed [15,16,17,18]. The high charge carrier mobility is the crucial factor in organic semiconducting materials for device applications and accordingly this topic has been covered in many articles, e.g., [15,16,17,18,19,20,21,22,23,24,25,26].

We have recently disclosed that the conducting properties of DLCs can be improved by the dispersion of various nanoparticles in DLC matrix. Due to technological applications and fundamental importance of these nanocomposites, the topic has received great deal of attention from scientists around the world that has been covered in many recent articles, e.g., [27,28,29,30,31,32,33,34,35,36,37]. In this article, we briefly present recent advances in the dispersion of small concentration of various nanomaterials in to columnar phases of DLCs. 

### 1.2. Nanomaterials

In recent year, there have been significant developments in the field of metallic, semiconducting and carbon nanostructure materials. These nanomaterials have found extensive commercial technological applications and also to comprehend different basic concepts at the nanometer range. Materials typically below 100 nm size in one-, two-, or all three-dimensions are defined as nanomaterials. They are made of inorganic as well as of organic materials and demonstrate physical characteristics quite different from those of individual particles or atoms and from mass materials [38,39]. Noticeable variation in electro-optical and biological properties has been seen as the span of size lessens to one billionth of a meter. The significant advantage of nanomaterials in different fields, for example, energy, computing, optics, catalysis, biomedical and biosensors have been widely investigated [38,39,40,41]. During the past two decades, nanoscience and nanotechnology saw colossal evolution, because of our capability to evaluate nano-sized materials with the assistance of present day instruments, such as a field emission scanning electron microscope (FE-SEM), an atomic force microscope (AFM), a scanning tunneling microscope (STM), and a high-resolution transmission electron microscopy (HR-TEM) etc. 

In this article, we mainly focused on recent advances in discotic liquid crystal-assisted nanoparticles (NPs). Combining nanomaterials in DLCs has been fast growing current hot research topic in which number of intriguing marvels have been demonstrated and accordingly such DLC-NPs hybrid systems may be useful for various device applications. We initiated this research program by dispersing various functionalized NPs in different DLCs [13,42,43,44,45,46,47,48,49,50,51,52,53,54,55,56,57,58,59,60,61,62,63,64,65,66,67,68,69,70,71]. Some aspects of DLC-NP composites are presented here. 

## 2. Nanoparticles in Discotic Liquid Crystals

### 2.1. Spherical or Quasi-Spherical Metallic NPs in Discotic Liquid Crystals

**Figure 1:**
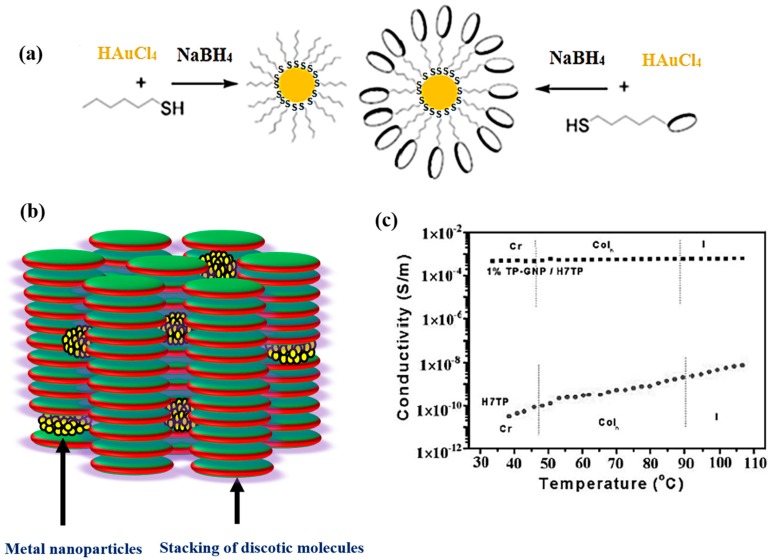
(**a**) Synthesis of alkanethiol and discotic functionalized GNPs; (**b**) Schematic illustration of dispersion of GNPs in discotic matrix; (**c**) Temperature variation of DC conductivity of pure and composite systems of GNPs. Reproduced with permission [45].

**Figure 2:**
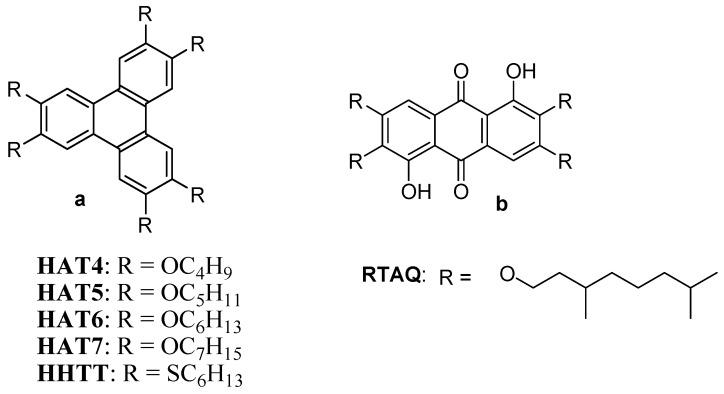
Molecular structures of commonly used triphenylene (**a**) and anthraquinone (**b**) based discotic liquid crystals.

The dispersion of nanoparticles in DLCs was initiated by Kumar and co-workers in 2004 [42]. Hexanethiol-functionalized gold nanoparticles (GNPs) of about 1.6 nm size were prepared (Figure 1) and dispersed in triphenylene-based DLCs (Figure 2) namely, hexahexylthiotriphenylene (HHTT), hexabutyloxytriphenylene (HAT4) and hexapentyloxytriphenylene (HAT5) by mixing 0.5% to 3% (by weight) [42,45]. Upon dispersion, the mesophase properties of discotics were not affected much, which were characterized by polarizing optical microscopy (POM), small angle X-ray scattering (SAXS) and differential scanning calorimetry (DSC) studies. A smooth dispersion of gold nanoparticles in mesophase was observed in low concentration; however, dispersion of larger amount leads to the aggregation of nanoparticles.

The doping of gold nanoparticles increases the electrical conductivity of the system significantly. Similarly, gold nanoparticles capped with triphenylene units were prepared, characterized and dispersed in a TP-based DLC namely hexaheptyloxytriphenylene (HAT7). Three composites 0.5 wt% TP-GNP/HAT7, 1 wt% TP-GNP/HAT7 and 2 wt% TP-GNP/HAT7 were prepared. All the composites show liquid crystalline behavior and display classical columnar phase texture upon cooling from isotropic liquid. The clearing temperatures of all the composites were decreases, as expected, upon increasing TP-GNP dispersion. TEM studies reveals that these TP-functionalized GNPs self-assembled into a hexagonal pattern on the surface. The electrical conductivity measurements were carried out while cooling form isotropic liquid for virgin HAT7 and 1 wt% TP-GNP/HAT7 nanocomposite. There is dramatic increasing conductivity by more than 10^6^ upon dispersion with 1 wt% TP-GNPs (Figure 1c). This enhancement to the electrical conductivity could be because of the formation of charge transfer complex between electron rich TP DLC and electron deficient GNPs (Figure 1) [45]. Holt et al. also reported similar enrichment in the conductivity upon doping with organic functionalized gold nanoparticles [22]. Similar results have been previously observed when 1 wt% of a strong electron deficient molecule TNF was doped in triphenylene DLCs. [14]. Shen et al. also prepared similar TP-protected GNPs and studied the self-assembly of these NPs on the surface [72]. The triphenylene ligands self-assembled in a stripe-like arrangement over gold nanoparticles surface via π–π interactions. They proposed that the spaces among triphenylene moieties on gold nanoparticles permit the inclusion of discotic TP molecules on neighboring GNPs to form a stripe-like assembly in a large area up to 0.5 μm. These nanocomposites provide a novel self-assembly structure for NPs and this linear 1D row in stripes have potential applications in electronics, as the 1 wt% doping with gold nanoparticle in discotic liquid crystals enhance the conductivity by 10^6^ fold.

To elucidate the optoelectronic properties of triphenylene-based DLC, Supreet et al. investigated the effect of dispersion of GNPs into mononitro–functionalized triphenylene discotic liquid crystal on their thermal, electrical and optical properties [55]. The dispersion of Au metal nanoparticles into the discotic columnar matrix decreases the orientational order parameter (S) and increases the relaxation time (τ). The effect of GNPs on dielectric properties was studied by Dhar et al. [56,65,67]. Different composites with wt% of GNPs with 0.2, 0.6 and 1.2 wt% were prepared in DLC. On increasing the concentration of GNPs in DLC, the plastic hexagonal columnar phase (Col_hp_)-isotropic transition temperature decreases but the crystal-Col_hp_ transition temperature does not alter much. Also, the lattice parameter decreases for the 1 wt% GNPs. Furthermore, the UV-Vis spectroscopy reveals that doping with GNPs in DLCs, changes the band gap (E_g_) of the system, which is reduced to 3.50 eV from 4.30 eV. This reveals “that the presence of GNPs influences forbidden energy gap of pure HAT4 and the conductivity has improved by the order of two to four” [56]. The composites show poor homeotropic alignment. The permittivity (ε^’^) of the composites was also enhanced for 0.2 wt% GNPs at the I-Col_hp_ transitions due to randomness of the discs in the Col_hp_ phase.

Recently, Basova et al. reported the dispersion of hexadecylamine coated gold nanoparticles (AuNP-HA) into nickel phthalocyanine DLCs (NiPcR_4_) [73]. Four composites of 0.1 wt% AuNP-HA/NiPcR_4_, 1 wt% AuNP-HA/NiPcR_4_, 2 wt% AuNP-HA/NiPcR_4_ and 5 wt% AuNP-HA/NiPcR_4_ are prepared and investigated for their thermal and electrical properties. Addition of nanoparticles of 0.1, 1, 2 and 5 wt% to the NiPCR_4_, decreases the columnar phase-isotropic phase transition temperature from 235 °C to 228 °C, 225 °C, 222 °C and 217 °C, respectively. The electrical conductivity of system enriched by more than two orders of magnitude.

We also investigated the self-assembled supramolecular structures of metal nanoparticles (gold and silver) in the columnar phase of metal free phthalocyanine DLCs [66]. The results clearly describe the uniform dispersion of NPs in columnar mesophase of phthalocyanine DLCs enhance the electrical conductivity of the nanocomposites up to four orders of magnitude without altering columnar phase. We also examined the nonlinear optical transmission of nanocomposites, when measured under excitation of nanosecond laser pulse at 532 nm. The enhancement of conductivity and NLO studies described in these nanocomposites makes them ideal materials for optoelectronics and photonics applications [66]. 

Recently, Yaduvanshi et al. [63] studied Ag nanoparticles size effects on HAT4 DLCs. The composites were made by wt% of 0.6% with the size of 6 nm and 100 nm. The result reveals that the 6 nm Ag NPs dispersion in DLC enhances the ionic conductivity as well as relative permittivity of the systems with an observation of relaxation mode. The band gap of the HAT4 also reduced from 4.2 eV to 3.3 eV. The mesomorphic and thermal properties of DLC are unchanged on dispersion of Ag NPs. The higher conductivity values obtained due to better homeotropic alignment in the case of small NPs compare to large NPs. The larger size 100 nm Ag NP DLC composite shows maximum dielectric strength i.e. δε_max_ = 0.96 at 130 °C with compare to pure HAT4 δε_max_ = 0.13 at 138 °C. We have demonstrated the dispersion of Ag nanoparticles in columnar phase of HAT4 and 1,5-dihydroxy-2,3,6,7-tetrakis(3,7-dimethyloctyloxy)-9,10-anthraquinone (RTAQ) DLCs at small concentration i.e., 0.5 wt%–3 wt%. The results show that the doping of a minute amount of AgNPs into a DLC host does not disturb the nature of the mesophase but a minor shift in the transition temperature is observed. The lattice parameter is reduced by 0.16 Å in HAT4 and 1.58 Å in RTAQ respectively, which enable more ordered molecular packing and compact structure and also core-core distance is reduced in RTAQ nanocomposites [68].

Furthermore, the effect of copper nanoparticles on HAT4 DLC with dispersion wt% of 0.6% was studied [60,61]. Again, the dispersion does not affect the mesomorphic property of columnar DLC. POM and DSC results revealed that the hexagonal pattern were retained but a minor increased to the clearing temperature were observed. The ionic conductivity of the composites was increased from 1.72 × 10^−9^ S·m^−1^ to 0.83 × 10^−7^ S·m^−1^ with change in the band gap from 4.2 eV to 3.3 eV. The optical study suggests that the surface plasmon resonance (SPR) has been introduced in the DLCs due to the incorporation of Cu NPs and the dielectric permittivity also increased. 

### 2.2. Quantum Dots in Discotic Liquid Crystals

Zero-dimensional quantum dots (QDs) are the quasi spherical NPs belong to the family of inorganic semiconducting NPs that have been broadly investigated during the past decade. Their significant applications in material and biological science have been well investigated [74,75,76,77]. A number of quantum dots, e.g., CdSe, CdS, CdTe, ZnO, ZnSe, PbS, PbSe, SnS, InP, InS, InN and so on, have been extensively considered for different physical properties. Cadmium selenide QDs have received much consideration of researchers globally in material science due to their interesting photoluminescence properties. The ultra-pure monodisperse Cd-based QDs were prepared and characterized by Murray et al. later various synthetic techniques have been utilized for their preparation [41,78,79,80]. Liquid crystalline mesophases can also be used to synthesis CdSe QDs [81]. With this prospect, the excellent optical and electronic property of CdSe QDs nanocomposites can be coupled with self-organizing behavior of LCs and such LC-QDs composites may exhibit different electro-optical properties. Dispersion of some QDs in calamitic liquid crystals has been studied [82]. We investigated the doping of CdSe and CdTe QDs into supramolecular order of DLC [51,58]. These organic soluble CdSe QDs were synthesized similar to reported method with some minor change in synthetic procedure and the obtained CdSe QDs were characterized by utilizing TEM, UV-Vis spectroscopy and photoluminescence spectroscopy. TEM result affirms the uniform size and shapes of CdSe QDs. Nanocomposites were prepared by blending both CdSe and CdTe QDs with triphenylene DLC in chloroform solvent under sonication and dried by removing solvent under vacuum. The thermophysical properties of these nanocomposites were analyzed by POM, DSC, XRD and DC conductivity studies. No significant change in POM, DSC and XRD was observed indicating that uniform dispersion of QDs in small amount without altering its liquid crystalline phase. Like GNPs doping with hexabutyloxytriphenylene DLC, the inclusion of CdSe and CdTe QDs into a columnar phase does not change crystal phase to columnar phase transition but it reduces the columnar phase to isotropic phase transition temperature. XRD studies confirm the hexagonal columnar phase of DLCs does not change in presence of small concentration of QDs. The QDs are commonly known for electron acceptors in organic-inorganic mixtures and this phenomenon is advantage for making hybrid polymer-inorganic solar cells [83]. Recently, dispersion of CdSe QDs into room temperature anthraquinone discotic liquid crystals were reported [64] and the results shows that, at low concentration (0.5 wt%), the thermal stability of columnar phase is increased. For higher concentration, QDs shows aggregates, decreases the stability of the mesophase. Similarly, conductivity is enriched by five orders of magnitude in case of lower concentration of QDs doped in RTAQ. However, for higher concentration (5 wt%), conductivity enhancement is poor. The insertion of CdSe and CdTe QDs in to DLCs demonstrate the enhancement in the conductivity compared to the pure compound. The enhancement in conductivity of nanocomposites could be accomplished because of interaction between electron donor ability of electron rich triphenylene unit and acceptor capability of inorganic semiconducting QDs. The improved conductivity makes QDs doped DLCs nanocomposites as active components for different device applications, for example, thin film transistors, LED’s and organic solar cell.

In general, it is seen that small size 0-D metallic or semiconducting NPs can be uniformly dispersed in small amount in to supramolecular order of DLCs. The doping of NPs increases the electrical conductivity of DLCs up to a million times under ambient conditions. This makes these nano-systems adequate for practical semiconducting applications.

## 3. One-Dimensional Nanostructures in Discotic Liquid Crystals

### 3.1. Gold Nanorods in DLCs

**Figure 3:**
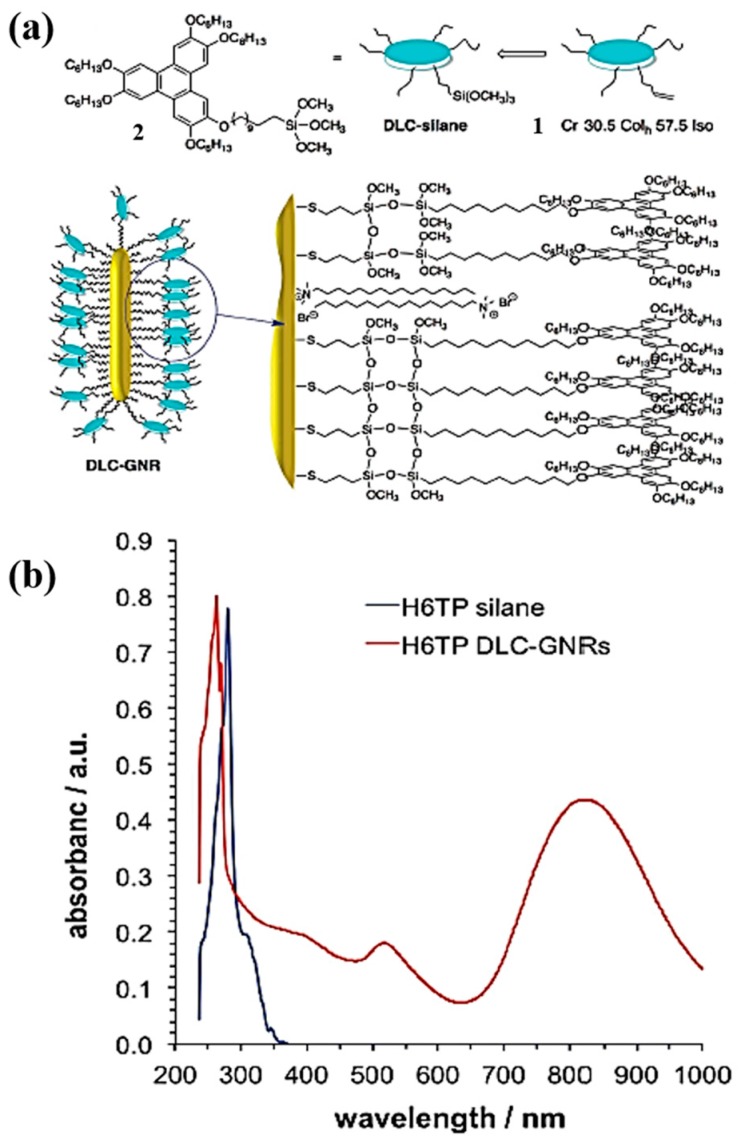
(**a**) Molecular structure of discotic mesogen bearing terminal silane precursor **2** and respective phase-transition temperature of the DLC silane precursor **1** and silane **2** along with 2D schematic representation of DLC-functionalized GNRs; (**b**) UV-Vis-NIR spectra of the TP DLC silane **2** and the TP DLC-GNRs. Reproduced with permission [91].

**Figure 4:**
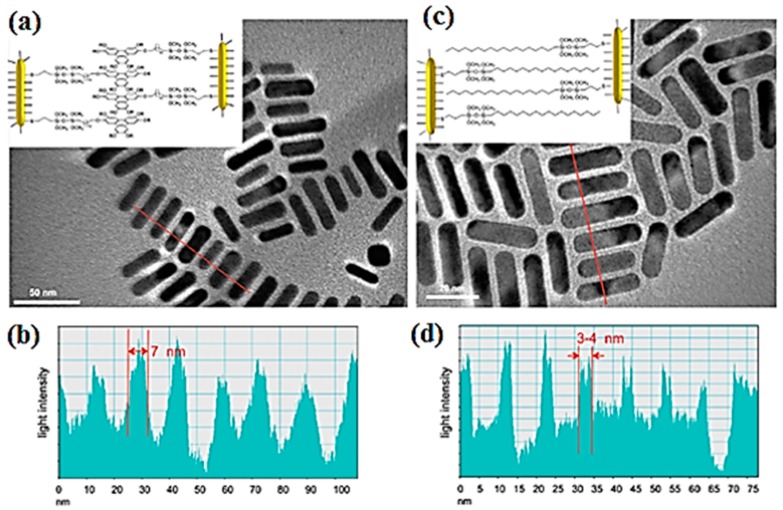
(**a**) TEM image of the DLC-GNRs; (**b**) Cross section profile of (**a**); (**c**) TEM image of the ODS-GNRs; (**d**) Cross section profile of (**c**). Insets show the arrangement of the GNRs in each case. Reproduced with permission [91].

Anisotropic one-dimensional nanostructures such as nanowires, nanorods, nanoribbons have many potential applications in optoelectronics, photovoltaics, sensors and memory devices. The effects of dispersion of elongated 1-D metallic nanostructures in liquid crystals have recently been reported [84,85,86,87]. We also investigated the effect of dispersion of gold nanorods into a soft matrix of a triphenylene derivative namely HAT5 [54]. Gold nanorods (GNRs) with an aspect ratio of 2.7 (15 nm wide and 40 nm long) were prepared via a seed-mediated growth method [88,89]. As prepared dodecanethiol passivated GNRs were dispersed in hexapentyloxytriphenylene via the solvent mixing method, as we have discussed earlier, in nanoparticles dispersion. The nanocomposites were studied using POM, SAXS, DSC, UV-Vis studies and DC conductivity studies. The nanocomposites show an increase in DC conductivity compared to the pure hexapentyloxytriphenylene DLC. Undoped hexapentyloxytriphenylene showed conductivity from 4.7 × 10^−10^ S·m^−1^ at 65 °C to 4.5 × 10^−9^ S·m^−1^ at 122 °C but in 1 wt% dispersion of GNRs in hexapentyloxytriphenylene increases the conductivity to 1.22 × 10^−6^ S·m^−1^ at 122 °C, also in 5 wt% GNRs-hexapentyloxytriphenylene nanocomposites it increases to the order of five. Such gold nanorods-hexapentyloxytriphenylene composites were further characterized to see the nanorods embedded discotic nanowire in dark field scanning transmission electron microscopy (STEM). It was reported that the addition of a polar solvent in a nonpolar solution of DLC forms discotic nanoribbons via π plans stacking [90]. Methanol was added to chloroform solution containing hexapentyloxytriphenylene and GNRs composites and forms discotic nanoribbons embedded with GNRs which was reveal in STEM characterization. Such molecular wires can be used in OFET, optoelectronics. Recently, Hegmann et al. reported the preparation and characterization, as well as the surface and bulk self-assembly, of GNRs functionalized with triphenylene-based discotic liquid crystal through silane conjugation [91] (Figure 3). The π–π interaction of triphenylene unit enhance the self-assembly DLC-GNRs form ribbons of several length. Further, compared with TEM images of ODS-coated GNRs with DLC-GNRs as shown in Figure 3. The TEM images obtained for DLC-GNRs composites as shown in Figure 4. The image Figure 4a and corresponding cross section profile Figure 4b shows that “the gap between DLC-GNRs is about 7 nm on average peak (widths were measured as the full width at half maximum), which matches perfectly with the length of the stacking molecules from neighboring GNRs with the triphenylene core overlapping each other (see inset, Figure 4a). For comparison, ODS-coated GNRs were also synthesized and a representative TEM image of the ODS-GNRs drop cast and dried from toluene is shown in Figure 4c. From the corresponding cross sectional profile (Figure 4d), the gap between the ODS-GNRs was measured to be 3–4 nm, which also matches with the molecular length of the alkyl chains of neighboring GNRs overlapping with one other.” The dispersion of host hexahexyloxytriphenylene (HAT6) DLCs with about 1 wt% by DLC-GNRs shows the reduction in the lattice parameter and intracolumnar packing. The high charge carrier mobility measurements of DLC-GNRs doped HAT4 (1 wt% DLC-GNR/HAT4 and 2 wt% DLC-GNR/HAT4), DLC-GNR-doped HAT6 (1 wt% DLC-GNR/HAT6) and undoped respective virgin discotic (HAT4 and HAT6) were investigated using the time-of-flight (TOF) techniques. The hole and electron mobilities of virgin HAT4 are 1.7 × 10^‒2^ cm^2^·V^‒1^·s^‒1^ and 2.0 × 10^‒2^ cm^2^·V^‒1^·s^‒1^ recorded in the Col_hp_ and the hole mobility of virgin HAT6 DLC is 2.0 × 10^‒4^ cm^2^·V^‒1^·s^‒1^ recorded in the Col_h_ phase. The hole and electron mobility of 1 wt% DLC-GNR/HAT4 and 2 wt% DLC-GNR/HAT6 was found to be 3.9 × 10^‒2^ cm^2^·V^‒1^·s^‒1^, 5.2 × 10^‒2^ cm^2^·V^‒1^·s^‒1^ and 0.7 × 10^‒2^ cm^2^·V^‒1^·s^‒1^, 2.4 × 10^‒2^ cm^2^·V^‒1^·s^‒1^. Overall, “the inherent charge carrier mobilities of both holes and electrons are comparable to those of pure HAT4 in the LC phase and marginally higher value of the mobility observed for the mixture of 1 wt% DLC-GNRs in HAT4” [91]. This has been attributed due to the optimal concentration of GNRs in HAT4. The sudden change in holes and electrons mobility in 2 wt% DLC-GNRs/HAT4 could be due to the onset aggregation of DLC-GNRs in the mesophase of HAT4 DLCs [91].

### 3.2. Carbon Nanotubes in DLCs

**Figure 5:**
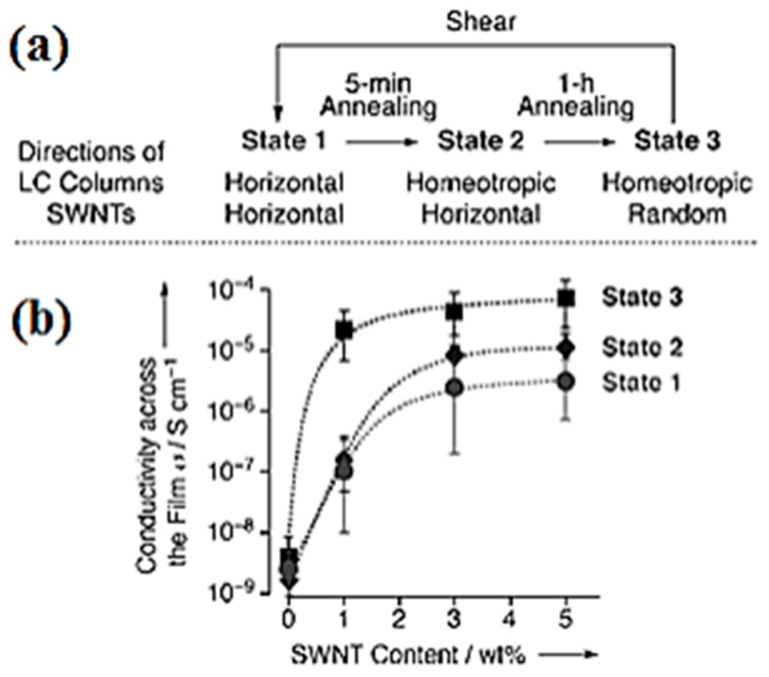
(**a**) Processing and orientational characteristics of states 1–3. ILC_col_/SWNT composites were sheared (state 1), shortly annealed for 5 min at 150 °C (state 2) and then annealed for 1 h at 150 °C (state 3). (**b**) Plots of conductivities across the film (s) of states 1–3 at 258 °C of ILC_col_ films doped with 0, 1, 3 and 5 wt% SWNTs, sandwiched by ITO electrodes with a separation of 12.5 μm. Reproduced with permission [98].

Carbon nanotubes (CNTs) have been well studied during the past few years because of their interesting electronic and physical properties [92,93,94,95,96,97]. The anisotropy, self-assembly, 1-D conducting properties of both CNTs and DLCs gathered much attention to see the structural-self-assembly properties of CNT-DLC hybrid systems and in this contest, we also investigated the inclusion of triphenylene and octadecylamine functionalized CNTs in TP based columnar mesophases [46,47]. Results indicated that at low concentration of CNT dispersion in columnar phases remain stable and CNTs are inserted in between discotic columns. In other words, CNTs can be aligned parallel to the columnar structure. In another study Lee et al. demonstrated that the molecular compatibility of discotic functionalized single-walled nanotubes (SWNTs) in triphenylene discotic stabilize the dispersion of CNTs and help the molecular orientation of CNTs [98]. SWNTs were dispersed in ionic liquid crystalline (ILCs) derivatives of TP. The π–π interactions of triphenylene molecules surrounding CNTs stabilize them in the director orientation. The “SWNTs are uniformly dispersed by a π-cation/π-electronic interaction and significantly form a 3D network structure associated along with an interionic interaction of ion based liquids.” The DC conductivity values of such composites were 3–4 times greater than undoped DLC. The conductivity values are completely dependent of the alignment or anisotropy of both LC columns as well as SWNTs to each other in electronic cell. After dispersion and annealing, composites were assembled in three states namely “state 1, where both LC columns and SWNTs were coaxially oriented horizontally with respect to the glass plates. State 2, where the LC columns were oriented homeotropically while maintaining the horizontal orientation of SWNTs, and state 3, where SWNTs were oriented randomly in the homeotropically oriented LC columns. This orientation of SWNTs is a dominant factor for the charge-carrier transport properties of the ILC_col_/SWNT composite. In DC conductivity values σ in the film is lower at room temperature in state 1 compare to stage 3, (Figure 5). The σ values in state 3 were 3–4 times greater than state 1, indicating SWNT orientation for better charge transport. The results reveal that considering ILC_cub_, compared with ILC_col_ accommodates SWNTs in its non-anisotropic cubic LC lattice” [98]. 

### 3.3. CdS Nanowires in DLCs

**Figure 6:**
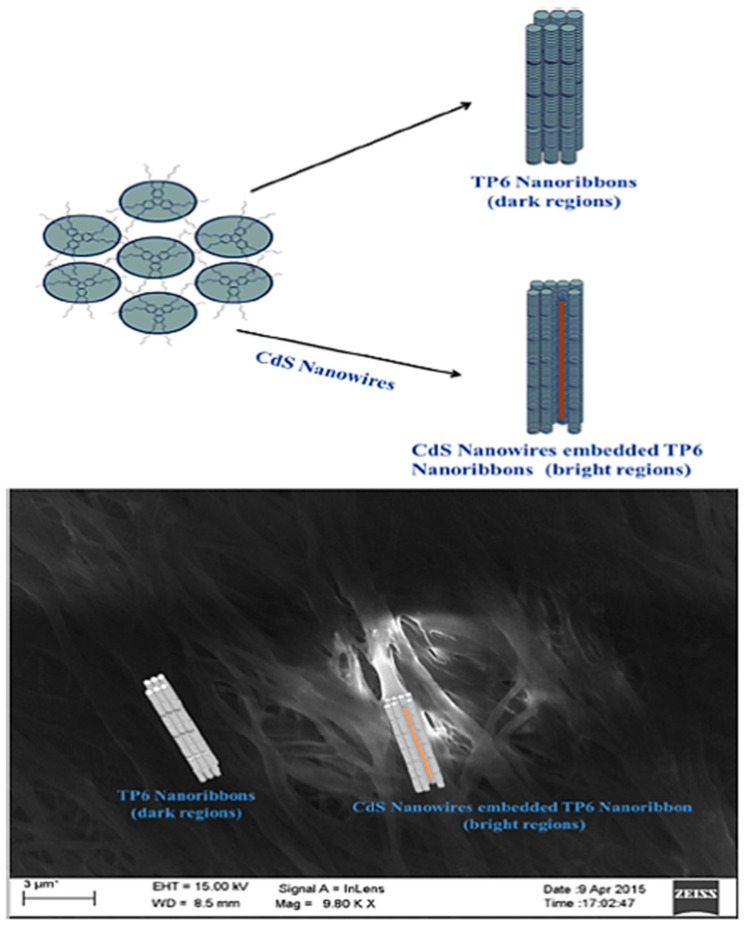
Schematic illustration of trapping of CdS nanowires in nanoribbons of triphenylene and respective FE-SEM images of 0.5 wt% CdS/TP6. Reproduced with permission [71].

Octylamine functionalized CdS nanowires were prepared, characterized and dispersed in the supramolecular order of a triphenylene-based DLC to see their effects on self-assembly and optical and thermal properties [71]. The two nanocomposites (0.5 wt% and 1 wt%) of CdS nanowires dispersed in hexahexyloxytriphenylene DLC were prepared by mixing CdS nanowires and DLC in chloroform. The nanocomposites were characterized by POM, DSC, XRD and SEM analysis. The POM textural observations clearly indicate the homogeneous dispersion of semiconductor nanowires in HAT6 DLC. There is a gradual decease in melting point on increasing the concentration of CdS nanowires. The presence of the CdS nanowires among the hexagonal self-assembly of triphenylene appears to induce slight disorder in alkyl chains of discotics at higher concentration of CdS nanowires and this contributes to decrease in the melting temperatures. The enthalpy of phase transition (∆*H*) also reduced significantly with increasing wt% of CdS nanowires, this again can be associated to nanoribbon induced disorder of alkyl chains. Presence of CdS nanowires among the hexagonal columnar phase induces a comparatively closer core-core packing of disc molecules observed in SAXS studies. It shows that the DC conductivity of the DLC increased by 3‒4 orders of magnitude upon doping with CdS nanowires. Based on experimental observation of POM, DSC, SAXS, SEM and EDXA, it is concluded that CdS nanowires get trapped in certain regions of ribbon like structures of triphenylene discotic liquid crystal. The general model depicted in Figure 6 shows self-assembly of hexahexyloxytriphenylene (TP6) nanoribbons with and without CdS nanowires. These hybrids of two different class of semiconductors makes themselves an important materials for devices like thin film transistors, LED’s and organic solar cell.

## 4. Two-Dimensional Nanostructures in Discotic Liquid Crystals

### 4.1. Graphene in Discotic Liquid Crystals

**Figure 7:**
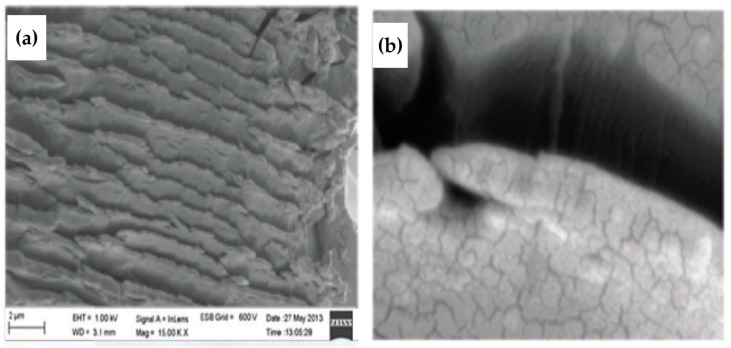
Cryo-SEM image of (**a**) octadecylamine functionalized graphene DLC composites showing a layered structure and (**b**) enlarged portion of (**a**) showing graphene layers inserted with discotics. Reproduced with permission [57].

Graphene, the one atom think, 2-D allotrope of carbon, has recently been extensively examined nanomaterial because of its fascinating exotic optical, electrical, mechanical, transport and thermal properties [99,100]. Graphene oxide (GO) obtained from chemical oxidation of graphite by Hummers methods [101]; later several reports appeared to modify it chemically for various applications [102]. Graphene might be considered as the largest polycyclic aromatic core. However, thermotropic LCs of graphene have so far not been realized but numerous studies have been reported on lyotropic LCs formed by GO. Recently we have published a comprehensive review [34] on ‘DLCs with graphene’ and, therefore only some important recent studies are presented here. We looked the dispersion of chemically functionalized graphene in discotic liquid crystals [57]. Carboxylic acid groups of GO were converted to more reactive acid chloride groups to react with octadecylamine (ODA). During this process, GO sheets were also reduced to yield ODA-functionalized reduced graphene oxide (RGO) which were fully characterized by various analytical tools like, IR spectroscopy, XPS (X-ray photon scattering), XRD studies, Raman spectroscopy and elemental analysis. The functionalized RGO was dispersed in a RTAQ DLCs. These nanocomposites were investigated by way of POM, DSC, SAXS and Cryo-SEM studies. Experiments indicate a sandwich like layered structure (Figure 7a,b). This propose that these discotic molecules are self-assembled in between f-graphene sheets. In other words, the nanocomposite system forms a “pillar-roof” kind of molecular arrangement where DLCs act as a pillars and graphene acts as a roof. The DC electrical conductivity studies of pure and nanocomposites show enhancement of electrical conductivity of the nanocomposites by three to four orders of magnitude. The ordered self-assembly of soft discotics molecules and f-graphene can be attributed to increasing electrical conductivity of the system.

On the other hand, surface functionalized graphene oxide with hexadecanethiol (HDT) and thiophenol (Tp) ligands destabilizes the mesophase [62]. The surface functionalization can be achieved by reacting the epoxy groups on the surface of the GO with thiols. Two nanocomposites of hexadecanethiol functionalized graphene oxide (1 wt% and 5 wt% HDT-GO/RTAQ) and thiophenol functionalized graphene oxide (1 wt% and 5 wt% Tp-GO/RTAQ) were prepared and characterized by POM, DSC, XRD and Cryo-SEM studies. These composites exhibit significant reduction in the isotropic transition temperatures and in the enthalpy of transitions. The disordered nature of the mesophase was also concluded from X-ray studies. However, these thiol-functionalized graphenes display intriguing properties of self-assembly on gold surfaces due to strong thiol-gold interactions. The self-assembly of monolayer on gold surface was affirmed by the combination of electrochemical, XPS, SEM and grazing angle IR studies.

**Figure 8:**
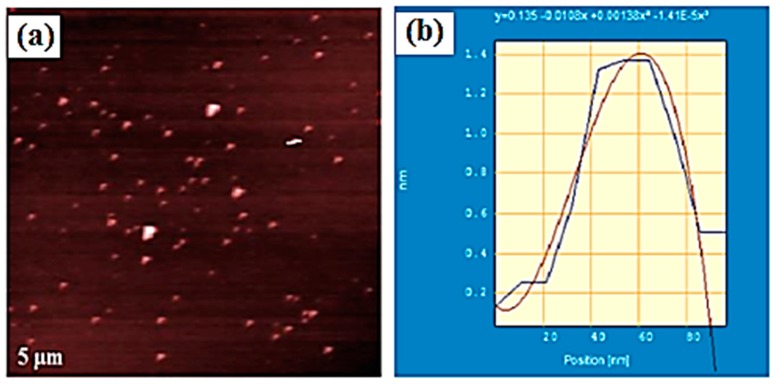
Morphology of graphene nanoparticles: (**a**) AFM image of f-rGONPs; (**b**) Height profile of f-rGONPs. Reproduced with permission [59].

We subsequently looked the doping of graphene nanoparticles (GrNPs) in DLCs [59]. GrNPs are ultra-thin few-layered pieces of graphene sheet with estimate size range of 50‒70 nm containing different thiols or on their edges and basal planes were functionalized with oxygen group. These functionalized reduced graphene oxide particles (f-rGOP) were synthesized (Figure 8) and dispersed in hexaalkoxy-triphenylene DLCs [59]. All the nanocomposites were characterized by POM, DSC, XRD, etc. techniques. The FE-SEM images reveal the uniform dispersion of NPs in the columnar phase of discotic LCs. The results indicate ordered self-assembly of f-rGONP and discotic molecules. The discotic molecules are adapted to self-assemble in sandwich likewise between functionalized graphene nanoparticles. The nanocomposite system shows enrichment of electrical conductivity by two-four orders upon doping with minute amount of f-rGONPs. This improvement in the conductivity is attributed due to the ordered orientation of f-rGONP and discotic molecules.

### 4.2. Metallic Nanodisks in Discotic Liquid Crystals

**Figure 9:**
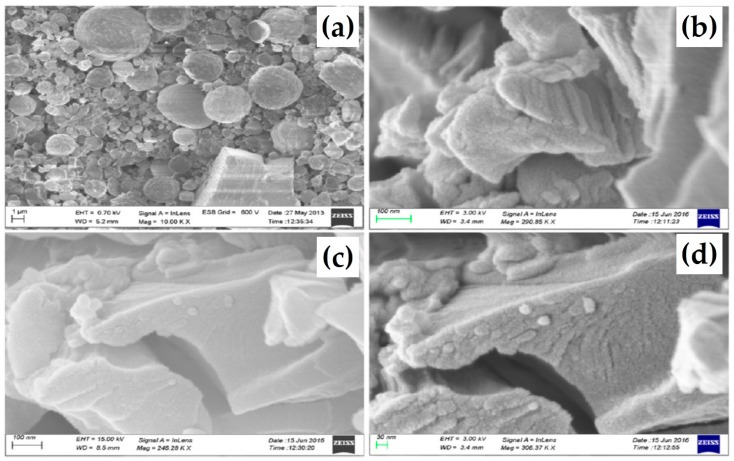
Cryo-SEM images of (**a**) parent RTAQ showing globular structures; (**b**,**c**) 3 wt% AgNDs/RTAQ in well pattered layer structures; and (**d**) enlarged portion of (**c**) showing bulges of nanodisks covered with discotic molecules. Reproduced with permission [70].

The morphology of metal or semiconductor nanomaterials has gained escalated consideration in recent years because of its impact on the physical and chemical properties. The opto-electrical properties of metal-nanostructures are dependent on their size, shape, dielectric properties, local environment, strong surface plasmon resonance, etc. The controlled synthesis of different dimensions, 0-D, 1-D and 2-D nanostructures have been extensively studied. We prepared 2-D silver nanodisks (AgNDs) in the presence of sodium bis(2-ethylhexyl) sulfosuccinate and functionalized using 1-hexanethiol ligand. The obtained AgNDs were characterized by UV-Vis spectroscopy, SAXS, FE-SEM, TEM and AFM studies. The presence of high aspect ratio of silver nanodisks (10–12 nm width, 30–35 nm diameter) were observed in SEM and TEM images. The parent silver nanodisk exhibit SPR band at 523 nm which is further shifted to lower region (10–12 nm) upon functionalized with alkane thiol. We have investigated the self-assembly of two dimensional thiol functionalized silver nanodisks dispersed in room temperature hexagonal columnar phase of RTAQ and HAT4 DLCs [70]. The nanocomposites of 0.5 wt% AgNDs/HAT4, 1 wt% AgNDs/HAT4, 3 wt% AgNDs/HAT4 and 3 wt% AgNDs/RTAQ were prepared and characterized using POM, DSC, SAXS, FE-SEM and AFM studies. In the nanocomposites, the lattice parameter values deceases by 0.16 Å which suggests that after doping the discotic molecules self-assembles in a “disk-in-discotic” like pattern by reducing the lattice parameter and this can also be further confirmed by Cryo-SEM self-assembly investigations. The studies reveal that the hexagonal columnar plastic lattice of HAT4 discotic does not change upon insertion of AgNDs and also the intercolumnar separation is not disturbed much. Similar diffraction values obtained for 3 wt% AgNDs/RTAQ nanocomposites. The core-core separation value is decreased by 0.03 Å and also reduction to the lattice parameter is observed at 40 °C. This reveals that the insertion of AgNDs forms compacted molecular structure which come closer by covering with DLC molecules. The Cryo-SEM results indicated the presence of layered structure due to stacking of nanodisks covered with DLC molecules which are absent in virgin DLCs (Figure 9). Further, the atomic weight percentage and the presence of the AgNDs are also characterized by energy dispersive X-ray analysis. The dispersion of these nanodisks increases the conductivity of the system by four-five orders of magnitude. 

## 5. Conclusions and Outlook

The LC nanoscience is budding up research field in soft matter science and has gained considerable attention during the past decade. The dispersion of metallic nanoparticles such as 0-D, 1-D and 2-D, carbon, metallic and semiconducting NPs in supramolecular order of discotic LC, which provides the materials that retains properties of nanomaterials along with self-organizing supramolecular architectures of DLCs, is currently a subject of great interest. A number of LC-nanomaterials hybrids have been synthesized and investigated their interesting physical properties, in addition to that, NPs is covalently linked to discotic mesogens have been synthesized in the liquid-crystalline medium itself. Efforts have been made to link DLCs covalently to NPs; in addition, various functionalized NPs have been doped in columnar phase of DLCs at room temperature or at elevated temperature. Despite the fact that NP-appended with discotic molecules do not show liquid crystalline properties in the virgin state, it has been noticed that small concentration of numerous NPs can be dispersed in the columnar matrix of DLCs. The 0-D NPs show random distribution; 1-D nanomaterials stay along the columns and 2-D nanomaterials lay on the surface of columns without disturbing their liquid crystalline properties. The inclusion of NPs in DLCs host imparts significantly effects on physical properties. The conductivity of the system increases by 2–6 orders of magnitude upon doping a minute number of various nanoparticles functionalized with aliphatic or aromatic ligands. Significant enhancement in the conductivity was observed when aromatic moieties functionalized nanoparticles are used. Thus, the dispersion of just 1% of triphenylene or thiophenol-functionalized gold nanoparticles improves the conductivity of the system by six orders of magnitude. However, only the bulk conductivity of the systems has been studied. The anisotropic nature of the conductivity upon doping nanoparticles has yet to be studied. As unlimited number of DLCs and NPs can be prepared, boundless hybrids are possible to investigate their physical properties. However, only limited investigations of DLC-NP composites have so far been done. Further detail investigations on opto-electronic properties, charge carrier mobility, alignment, anisotropic behavior, etc., need to be explored to utilize them in various devices.
